# Distal transradial access for percutaneous coronary intervention: a single-center randomized controlled study (DRAGON study)

**DOI:** 10.1186/s12872-025-05456-3

**Published:** 2025-12-23

**Authors:** Minghao Liu, Yuan Du, Cheng Cui, Jinwei Zhai, Wei Yu, Hao Zhang, Hongmei Liu, Xiongwei He, Zhenxi Shao, Yana Tong, Yan Liu, Xiaoyang Song, Na Wang, Ang Li, Fujian Duan, Hui Li, Yiying Song, Can Li, Yang Yang, Ying Song, Lei Song, Jue Chen, Jinqing Yuan, Weixian Yang, Yongjian Wu, Zhan Gao, Huanhuan Wang, Lijian Gao

**Affiliations:** 1 Department of Cardiology, Fuwai Hospital, Chinese Academy of Medical Sciences, Beijing, China; 2 Department of Cardiology, Qinhuangdao Third Hospital, Qinhuangdao, Hebei Province China; 3 Department of Cardiology, The Eighth Division Shihezi General Hospital (Shihezi People’s Hospital, The Third Affiliated Hospital of Shihezi University Medical School), Shihezi, Xinjiang Uygur Autonomous Region China; 4 Interventional Catheterization Laboratory, Fuwai Hospital, Chinese Academy of Medical Sciences, Beijing, China; 5 Department of Ultrasound Imaging, Fuwai Hospital, Chinese Academy of Medical Sciences, Beijing, China; 6 Coronary Heart Disease Center, Department of Cardiology, Fuwai Hospital, Chinese Academy of Medical Sciences, No.167 North Lishi Road, Xicheng District, Beijing, China

**Keywords:** Distal transradial access, Percutaneous coronary intervention, Transradial access, Postoperative hand function recovery

## Abstract

**Background:**

Transradial access (TRA) is the standard vascular approach for coronary interventions because it lowers bleeding risk and improves patient comfort. Nevertheless, radial artery occlusion (RAO) remains an important limitation, preventing re-use of the radial artery for subsequent procedures. Distal transradial access (dTRA) has been proposed as a strategy to further reduce RAO while preserving procedural efficacy.

**Aims:**

To assess whether dTRA reduces early and mid-term RAO compared with conventional TRA in patients undergoing percutaneous coronary intervention (PCI).

**Methods:**

In this single-center, prospective, randomized, open-label superiority trial, 426 consecutive patients scheduled for elective PCI between July 2023 and January 2024 were assigned 1:1 to dTRA (*n* = 213) or TRA (*n* = 213). Duplex ultrasonography was used to assess RAO at 24 h and 30 days. Primary endpoint: 24-hour RAO; secondary endpoints: 30-day RAO, procedural success, hemostasis time, radiation exposure, and hand/wrist function scores.

**Results:**

One patient withdrew consent, leaving 425 patients (dTRA 213, TRA 212) for analysis. The 24-hour RAO rate was significantly lower in the dTRA group compared to the TRA group (0.5% vs. 8.7%, *p* < 0.001). At 30 days, RAO remained lower in the dTRA group (1.6% vs. 8.2%, *p* < 0.001). Median hemostasis time was 3.0 h with dTRA versus 6.0 h with TRA (*p* < 0.001). Procedural success was comparable between groups (82.2% vs. 80.2%; *p* = 0.72), as was the fluoroscopy dose (1630.0 mGy [1069.0–2481.0] vs. 1598.0 mGy [1039.0–2512.0], *p* = 0.73). Early wrist function scores were marginally better in the dTRA group (*p* = 0.048).

**Conclusions:**

In elective PCI patients, dTRA significantly reduces RAO incidence and shortens hemostasis time compared to TRA, without compromising procedural success or increasing radiation exposure, while modestly improving early hand function. These data support routine adoption of dTRA for coronary interventions.

**Trial registration:**

Chinese Clinical Trial Registry: ChiCTR2300073902 (registered on 25 July 2023).

**Supplementary Information:**

The online version contains supplementary material available at 10.1186/s12872-025-05456-3.

## Introduction

The transradial access (TRA) approach has become the preferred method for percutaneous coronary interventions (PCI) worldwide. TRA offers high procedural efficacy and an excellent postoperative safety profile, and is therefore recommended by major professional societies, including the Society for Cardiovascular Angiography and Interventions (SCAI) and the European Society of Cardiology (ESC) [1, 2]. 

However, with the increasing adoption of TRA, certain complications and issues related to patient comfort have drawn attention. Among these, radial artery occlusion (RAO) remains one of the most common complications. Reported RAO rates vary across studies but consistently reflect a concerning frequency: for instance, a 2021 randomized controlled trial (RCT) by Eid-Lidt et al. reported RAO rates of 8.4% at 24 h and 5.6% at 30 days [3]. Another study by Fan et al. found 1-year RAO rates of 8.57% and 12.84% when 6F and 7F sheaths were used, respectively [4]. Furthermore, a meta-analysis showed in-hospital RAO rates of 5.93% (203/3,426) and a maximum follow-up RAO rate of 5.95% (279/4,690) [5]. 

Although most cases of RAO are clinically silent, imaging studies have demonstrated reduced blood flow to the thumb dorsum [6]. This complication can also impede future PCI procedures, radial artery grafting for coronary artery bypass surgery, and arteriovenous fistula creation for dialysis. Beyond RAO, clinical practice has revealed additional comfort and functional issues associated with TRA. Although shortening radial artery hemostasis times can reduce RAO rates [7], the recommended hemostasis time of under 2 h is difficult to achieve in most Chinese centers, where standard hemostasis durations are 6 h or longer. Prolonged hemostasis can cause hand swelling, numbness, and restricted wrist mobility. For patients requiring left-hand access, maintaining a pronated position during the procedure also results in significant discomfort.

Since 2017, when Kiemeneij et al. first introduced distal transradial access (dTRA) for coronary interventions, many experts and teams have explored its feasibility and safety [8]. Multiple clinical studies have highlighted the significant advantages of dTRA in preventing RAO, with rates consistently below 5%, and in some cases under 1% [5]. Additionally, a large multicenter prospective study from Korea, which included 4,977 patients, reported a combined RAO rate of 0.8% for radial and distal radial access [9]. Meanwhile, large-scale studies and meta-analyses have confirmed a lower incidence of hematoma at the puncture site with dTRA [10]. 

Despite the advantages of dTRA, its adoption remains limited due to unresolved challenges and insufficient clinical evidence in certain areas. Key issues include: (1) Most existing dTRA studies include both coronary angiography and PCI patients, yet the longer procedure duration, higher heparin doses, and prolonged hemostasis times required for PCI patients may affect RAO outcomes. The specific impact of dTRA versus TRA in this patient group remains unclear. (2) The effectiveness of dTRA for PCI, including procedural success rates and device choices, requires further evidence. (3) dTRA is technically demanding, often requiring longer puncture times and higher radiation doses during initial attempts, though the long-term impact of these factors on hospitalization duration and costs is uncertain [9, 11]. (4) While some studies have assessed pain associated with dTRA, there is little research on postoperative hand and wrist function [12]. 

To address these gaps, we designed and conducted this prospective, randomized controlled trial to evaluate the safety and efficacy of dTRA for PCI, including its impact on postoperative functional recovery and hospital costs.

## Methods

### Study design and population

This study was a single-center, prospective, randomized, superiority, open-label trial conducted at Fuwai Hospital, Beijing, China. Consecutive patients diagnosed with coronary artery disease and scheduled for percutaneous coronary intervention (PCI) were screened between July 2023 and January 2024. Participants were randomized in a 1:1 ratio to undergo PCI via dTRA or conventional TRA. Enrollment continued until the target sample size was fully achieved according to the pre-specified protocol, and no interim analysis was performed.

**Fig. 1:**
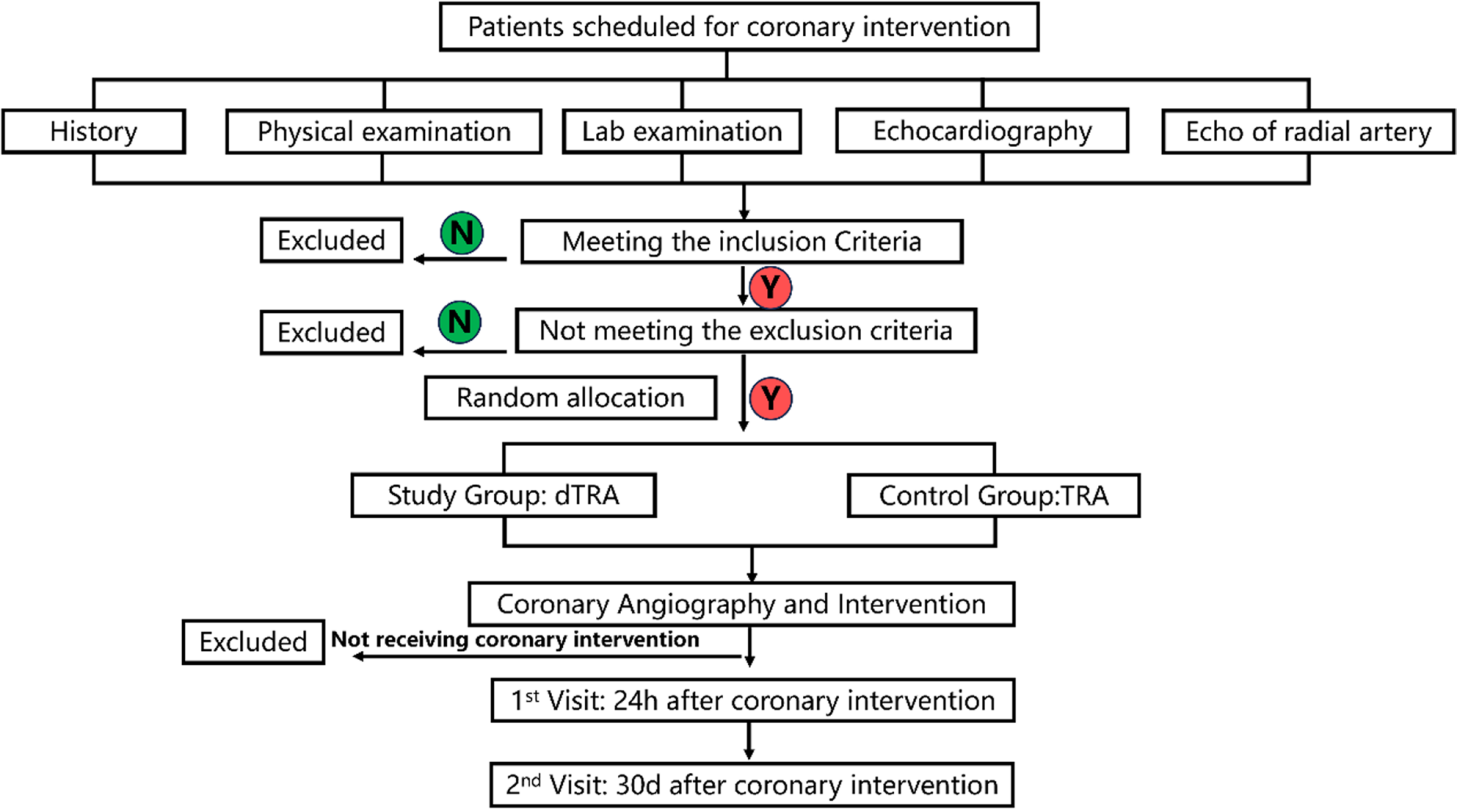
The flowchart of the study. Y = Yes, N = No, dTRA = distal transradial access, TRA = Transradial access

The study protocol has been previously published and is available for detailed reference [13]. The trial was registered with the Chinese Clinical Trial Registry under registration number ChiCTR2300073902 (registered on 25 July 2023). The flow chart was seen in Fig. 1. This trial was conducted and reported in accordance with the CONSORT 2010 Statement and the checklist has been uploaded as Supplemental Appendix S1.

### Randomization

Randomization was performed using a centralized electronic randomization system managed by the Center for Medical Statistics at Fuwai Hospital. Patients were stratified by diabetes status and ticagrelor use to ensure balanced distribution of these potential confounders between groups. The randomization results were concealed before patient enrollment and baseline assessment to minimize allocation bias.

### Inclusion and exclusion criteria

The inclusion criteria were as follows:

- Age between 18 and 80 years.
- Inner diameter of the distal radial artery ≥ 1.7 mm, confirmed by ultrasound.
- Positive Allen test results, with unobstructed radial artery blood flow.
- No prior interventions or surgeries involving the radial artery on the operative side.
- Scheduled for PCI and consent to participate in the study.

The exclusion criteria included:

- Acute myocardial infarction requiring immediate direct PCI.
- Occlusion of the radial artery on the operative side, confirmed by ultrasound.
- Negative Allen test.
- History of bilateral radial artery intervention or surgery.
- Significant renal insufficiency (eGFR < 30 mL/min).
- Allergy or intolerance to antiplatelet therapy.
- Patient refusal to participate.

### Procedural techniques

#### Radial access

Participants in the dTRA group underwent arterial puncture at either the anatomical snuffbox or the Hegu acupoint, while those in the TRA group underwent puncture at the conventional radial artery location, 2–3 cm proximal to the wrist crease. Preoperative ultrasound was used to localize the target vessel and confirm suitability for puncture. All procedures were performed by experienced interventional cardiologists proficient in both techniques.

A 6F arterial sheath (Abbott, USA) was used for most procedures, with thin-walled 7F sheaths (APT Medical, China) employed when clinically necessary. Heparin was administered at 100U/kg prior to PCI, with additional doses based on procedural duration (over 1 h) and activated clotting time (ACT) monitoring. Procedural details, including sheath insertion, contrast volume, and radiation dose, were recorded.

#### Hemostasis protocol

Referring to the approach used in the PROPHET trial, hemostasis was performed using the “patent hemostasis” protocol, defined as maintaining antegrade radial artery flow during compressive hemostasis to maximize the prevention of radial artery occlusion [14]. In the dTRA group, hemostasis was achieved using elastic bandage compression, which was removed after 3 h if bleeding had ceased. After applying the bandage, simultaneously monitor the patient’s proximal radial artery pulse, ulnar artery pulse, thumb color, and pulse oximetry waveform of the thumb. In cases where bleeding or mild hematoma formation occurred upon bandage adjustment, compression was reapplied for an additional 30–60 min. For the TRA group, hemostasis was achieved using a spiral artery compressor. During the initial hemostasis, the presence of a radial pulse distal to the compression site was confirmed. The device was gradually loosened every 2 h to assess radial artery pulsation and was fully removed after 6 h, ensuring uninterrupted blood flow throughout the hemostasis process. Hemostasis times and any bleeding-related complications were recorded for all participants.

#### Evaluation of the radial artery

The first and second follow-up times were 24 h and 30 days post-procedure, respectively. Radial artery ultrasound results and vascular access-related complications were recorded. The flow chart was seen in Fig. 1.

### Outcomes

The primary endpoint was the incidence of RAO at 24 h post-procedure, assessed via ultrasound. RAO was defined as the absence of detectable blood flow in the radial artery.

Secondary endpoints included RAO incidence at 30 days post-procedure, access crossing, procedural success rates, defined as the successful completion of PCI, hemostasis time, postoperative hand function recovery, evaluated using the Fugl-Meyer motor function scale [15] (Suppl Table 1) and safety outcomes, including hematoma, bleeding events, and neurological complications.

### Data collection, storage, and management

All data were collected by trained research nurses and entered into a secure electronic data capture (EDC) system compliant with Good Clinical Practice (GCP) guidelines. The EDC system featured built-in validation rules to prevent data entry errors and audit trails to track all changes. Data were de-identified, encrypted, and stored on a secure server with restricted access. Only authorized study personnel had access to the database, and all identifiable information was removed prior to analysis to ensure participant confidentiality.

### Statistical analysis

The primary analysis was conducted on the full analysis set (FAS) based on the intention-to-treat (ITT) principle. The FAS included all randomized patients who provided valid data for at least one outcome measurement post-intervention. Per-protocol (PPS) and as-treated (ATS) analyses were performed as sensitivity analyses. The PPS excluded patients with major protocol violations, ensuring a stricter analysis of patients who adhered closely to the study protocol. The ATS analyzed patients according to the access site actually used, regardless of their original randomization group. Continuous variables were presented as mean ± standard deviation (SD) or median with interquartile range (IQR) and compared using Student’s t-test or Mann-Whitney U test, as appropriate. Categorical variables were expressed as frequencies and percentages and compared using the chi-square or Fisher’s exact test.

The primary endpoint was analyzed using the chi-square test, with effect sizes reported as risk differences and 95% confidence intervals. Missing data were handled using multiple imputation techniques or worst-case imputation, depending on the variable’s relevance to primary or secondary outcomes.

Sample size calculations were based on prior literature indicating expected 24-hour RAO rates of 8.8% for TRA and 1.2% for dTRA. With a power of 90% and a two-sided significance level of 0.05, a total of 428 patients (214 per group) was required to detect a statistically significant difference, accounting for a 10% dropout rate.

All analyses were performed using SAS version 9.4 (SAS Institute, Cary, NC, USA). A *p*-value < 0.05 was considered statistically significant.

### Ethical considerations

The study was approved by the Ethics Committee of Fuwai Hospital, Chinese Academy of Medical Sciences & Peking Union Medical College (Approval No. 2023 − 1931). Informed consent was obtained from all participants prior to enrollment. The study adhered to the principles of the Declaration of Helsinki and Good Clinical Practice guidelines.

## Results

### Study population and baseline characteristics

**Fig. 2:**
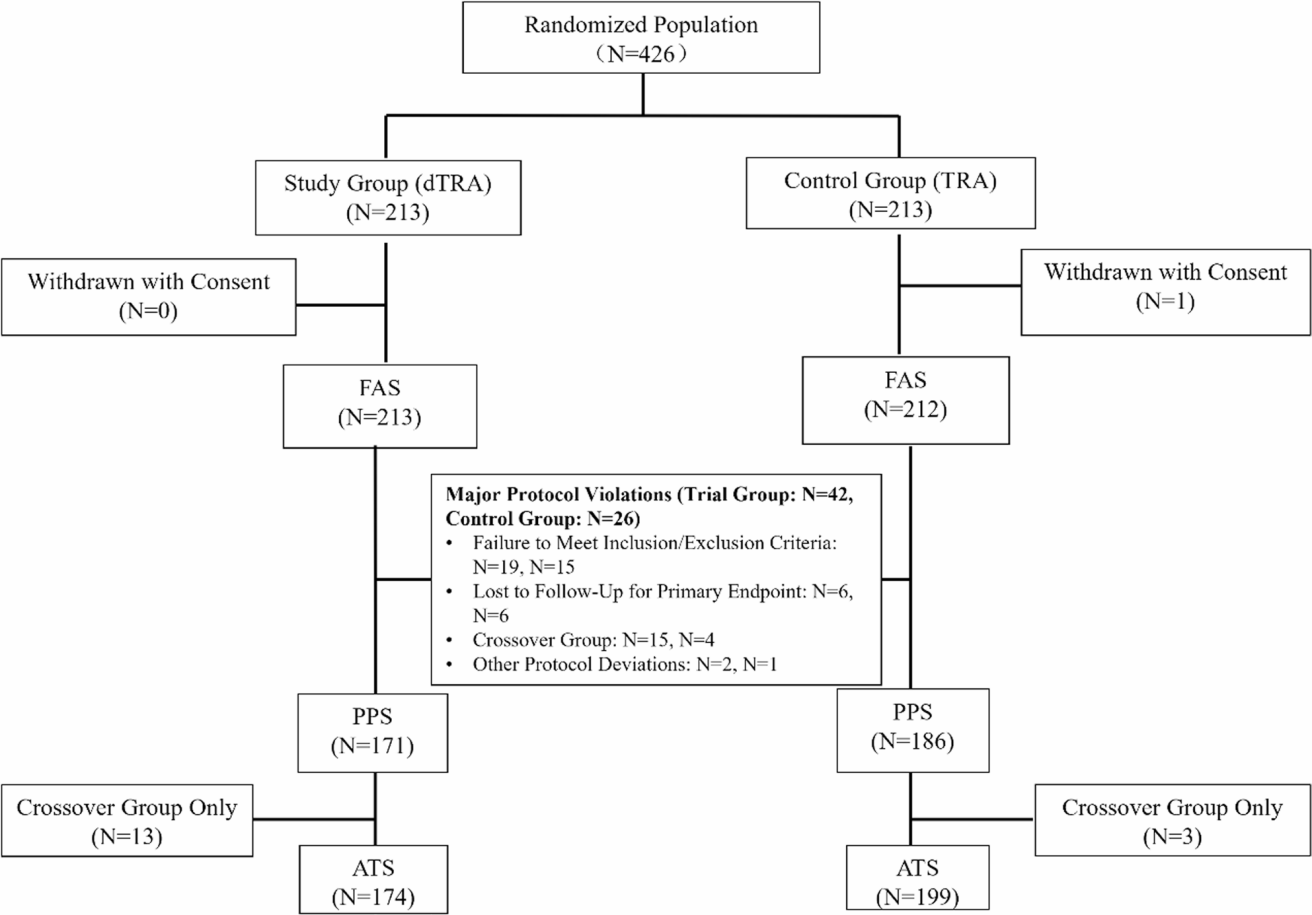
Schematic Diagram of Participant Randomization and Final Enrollment Status. dTRA = distal transradial access, TRA = Transradial access, FAS = Full Analysis Set, PPS = Per Protocol Set, ATS = As-Treated Set

A total of 426 patients were randomized in this study, with 213 patients allocated to the dTRA group and 213 to the TRA group. One patient in the TRA group withdrew consent after randomization, resulting in a final study population of 425 patients (dTRA: 213; TRA: 212), which formed the FAS. The composition of the datasets used for analysis is described below (Fig. 2).

A total of 68 patients were excluded from the per-protocol analysis for the following reasons: protocol violations (dTRA: 19; TRA: 15), loss to follow-up for the primary endpoint (dTRA: 6; TRA: 6), crossover to an alternate access site (dTRA: 15; TRA: 5), and other deviations from the study protocol (dTRA: 2; TRA: 1). After these exclusions, the PPS comprised 171 patients in the dTRA group and 186 patients in the TRA group. This dataset was used for sensitivity analyses to verify the robustness of the findings from the FAS.

The ATS analyzed patients according to the access site actually used, regardless of their original randomization group, thereby accounting for crossover between groups. During the procedure, 13 patients in the dTRA group switched to TRA, and 3 patients in the TRA group switched to dTRA. Based on the access route actually used, the ATS comprised 174 patients in the dTRA group and 199 patients in the TRA group.

Baseline demographic and clinical characteristics, including age, sex, body mass index, coronary disease classification, comorbidities (e.g., hypertension, diabetes, hyperlipidemia, etc.), prior revascularization history, and medication use, were well balanced between the two groups, with no statistically significant differences observed. Echocardiographic parameters were also comparable between groups (Table 1).

**Table 1:** Analysis results of demographic data and clinical characteristics during the screening period of participants

	dTRA Group (*N* = 213)	TRA Group (*N* = 212)
*Baseline demographic data*		
Age (y.o)	62.54, 53.13–68.37	63.41, 53.96–68.32
Gender (M/F)	170/43	160/52
Weight (kg)	75.00, 67.00–80.00	71.00, 64.00–80.00
Height (cm)	170.00, 166.00-175.00	170.00, 164.00-175.00
Body Mass Index (kg/m²)	25.89, 23.84–27.68	25.25, 23.14–27.76
Smoking (N, %)	116, 54.5%	115, 54.2%
Drinking (N, %)	43, 21.2%	46, 21.7%
*Comorbidities (N*,* %)*		
Hypertension	140, 65.7%	135, 63.7%
Diabetes	93, 43.7%	84,39.6%
Hyperlipidemia	180, 84.5%	174, 82.1%
Arrhythmia	15, 7.0%	11, 5.2%
Cerebrovascular Disease	21, 9.9%	21, 9.9%
Chronic Kidney Disease	0, 0	2, 0.9%
History of Myocardial Infarction	51, 23.9%	51, 24.1%
History of PCI/CAG	102, 47.9%	100, 47.2%
History of CABG	2, 0.9%	2, 0.9%
*Medication Use (N*,* %)*		
Aspirin	209, 98.1%	205, 97.2%
Clopidogrel	187, 87.8%	177, 83.9%
Ticagrelor	23, 10.8%	30, 14.2%
Indobufen	2, 0.9%	4, 1.9%
Low-Molecular-Weight Heparin	0, 0	3, 1.4%
Tirofiban	1, 0.5%	0, 0
Rivaroxaban	3, 1.4%	5, 2.4%
Nitrates	146, 68.5%	153, 72.5%
Calcium Channel Blockers	42, 19.7%	41, 19.4%
*Echocardiographic parameters*		
LVEDD (mm)	47.00, 45.00–50.00	47.00, 44.00–50.00
LVEF (%)	64.00, 60.00–65.00	63.00, 60.00–65.00
LA diameter (mm)	37.00, 34.00–39.00	36.00, 34.00–39.00

### Coronary lesion characteristics and procedural outcomes

#### Coronary lesion characteristics

The characteristics of coronary lesions were similar between the dTRA and TRA groups, ensuring baseline comparability. Patients in both groups exhibited single-vessel, double-vessel, and triple-vessel disease, with double-vessel disease being the most common presentation, followed by triple-vessel and single-vessel disease.

Lesion complexity, assessed using the ACC/AHA classification system, was comparable between the groups. The majority of patients presented with complex Type C lesions, followed by Type B and Type A lesions.

Special types of coronary lesions, including chronic total occlusions (CTO) and in-stent restenosis (ISR), were also evenly distributed. Specifically, 48 patients in the dTRA group and 47 patients in the TRA group had CTOs, while 14 patients in the dTRA group and 13 patients in the TRA group presented with ISR.

The Syntax score, a quantitative measure of lesion complexity and severity, was also similar between the groups. The mean Syntax score in the dTRA group was 12.00 (7.00–20.50), while in the TRA group, it was 13.00 (7.00–20.00). (Table 2).

**Table 2:** Analysis results of coronary lesion characteristics

	dTRA Group (*N* = 213)	TRA Group (*N* = 212)
*Number of coronary vessels with stenosis (N*,* %)*		
Single-vessel disease	40, 18.8%	50,23.6%
Double-vessel disease	68,31.9%	64, 31.2%
Triple-vessel disease	81, 38.0%	76, 35.8%
Left main disease	4, 1.9%	1, 0.5%
Left main + single-vessel disease	1, 0.5%	6, 2.8%
Left main + double-vessel disease	5, 2.3%	7, 3.3%
Left main + triple-vessel disease	11, 5.2%	5, 2.4%
No Abnormalities	3, 1.4%	3, 1.4%
*ACC/AHA classification of coronary artery disease (N*,* %)*		
Type A lesions	13, 6.1%	16, 7.5%
Type B lesions	36, 16.9%	34, 16.0%
Type C lesions	161, 75.6%	159, 75%
Normal	3, 1.4%	3, 1.4%
*Special types of coronary lesions*		
Chronic Total Occlusions (CTO), (N, %)	48, 22.5%	47, 22.2%
In-stent Restenosis (ISR), (N, %)	14, 6.6%	13, 6.1%
Syntax score	12.00, 7.00-20.50	13.00, 7.00–20.00

### Procedural outcomes

The procedural success rate, defined as effective revascularization of the target lesion, was high and comparable between the two groups. Of the 425 patients included in the full analysis set (FAS), a total of 380 patients underwent PCI: 187 in the dTRA group and 193 in the TRA group. This represented a PCI rate of 87.8% in the dTRA group and 91.0% in the TRA group, with no significant difference between the groups (*p* = 0.276). Among these patients, the procedural success rate was 93.6% in the dTRA group and 88.1% in the TRA group (*p* = 0.076).

In terms of interventional techniques, the number of stents implanted was similar between the groups. A total of 231 stents were deployed in the dTRA group, compared to 226 in the TRA group. There were no significant differences in the use of other interventional techniques, such as balloon angioplasty without stenting. Neither group required advanced techniques, including rotational atherectomy, intravascular lithotripsy, or laser atherectomy.

The mean number of puncture attempts was higher in the dTRA group compared to the TRA group (1.38 ± 0.94 vs. 1.18 ± 0.51, *p* < 0.001). Median puncture time was 1.0 min (1.0–3.0) in the dTRA group and 1.0 min (1.0–2.0) in the TRA group, with no statistically significant difference (*p* = 0.200). Procedural time, radiation exposure, and contrast volume were all comparable between the two groups. The median procedural time was 35.00 min (25.00–51.00) in the dTRA group and 40.00 min (25.00–56.00) in the TRA group, with no statistically significant difference between the two approaches (*p* = 0.803). Similarly, radiation exposure was similar between groups, with median doses of 1,630.0 mGy (1,069.0–2,481.0) in the dTRA group and 1,598.0 mGy (1,039.0–2,512.0) in the TRA group (*p* = 0.734). The median contrast volume was identical in both groups, at 170.0 mL (150.0–180.0, *p* = 0.421) (Table 3).

**Table 3:** Procedural outcomes of participants

	dTRA Group (*N* = 213)	TRA Group (*N* = 212)	*P* Value
*Procedural outcomes (N*,* %)*			
PCI rate	187, 87.8%	193, 91.0%	0.276
PCI success rate	175, 93.6%	170, 88.1%	0.076
*Interventional techniques (N*,* %)*			
Stent implantation	151, 80.7%	148,76.7%	0.960
Balloon angioplasty	7, 3.7%	11, 5.7%	
Drug-eluting balloon angioplasty	14, 7.5%	13, 6.7%	
Number of Stents	231	226	0.451
*Puncture technique*,* radiation dose*,* and contrast agent usage*			
Number of puncture attempts	1.38 ± 0.94	1.18 ± 0.51	0.010
Puncture time (min, IQR)	1.0, 1.0–3.0	1.0, 1.0–2.0	0.225
*Study procedure side (N*,* %)*			0.096
Right	100, 46.9%	82, 38.7%	
Left	113, 53.1%	130, 61.3%	
Procedural time (min, IQR)	35.00, 25.00–51.00	40.00, 25.00–56.00	0.803
Radiation exposure (mGy, IQR)	1630.0, 1069.0-2481.0	1598.0, 1039.0-2512.0	0.734
Contrast volume (mL, IQR)	170.00, 150.00-180.00	170.00, 150.00-180.00	0.421

### Procedural safety

Minimal access-related complications were reported in both dTRA and TRA groups. Minor hematomas occurred in one patient in each group, graded as EASY I in the dTRA group and EASY II in the TRA group. No cases of pseudoaneurysm, arteriovenous fistula, or major bleeding (BARC grade 2 or higher) were reported in either group.

While no serious adverse events related to the vascular access site occurred in the dTRA group, two significant events were recorded in the TRA group. One patient experienced suspected heparin resistance, leading to coronary thrombosis during the procedure. This patient required emergent extracorporeal membrane oxygenation (ECMO) and intra-aortic balloon pump (IABP) placement but eventually withdrew consent and left the hospital. Another patient in the TRA group experienced coronary perforation, resulting in cardiac tamponade. This required urgent pericardiocentesis and additional stent placement, but the patient recovered and was successfully discharged.

### Primary endpoint: radial artery occlusion at 24 h

**Fig. 3:**
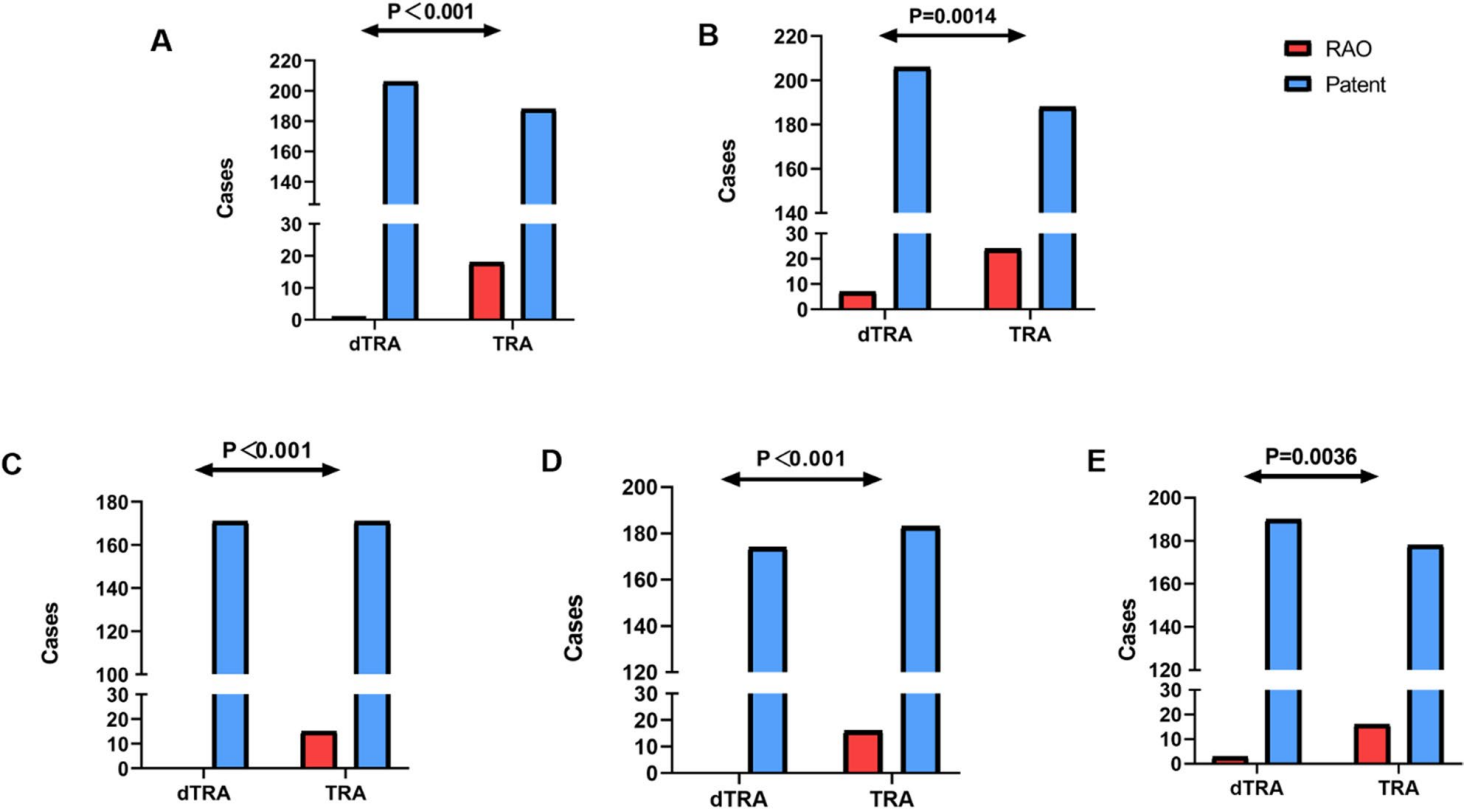
Statistical Analysis Results of Radial Artery Occlusion at 24 h Postoperatively (**A**-**D**) and 30 Days (**E**). **A** shows the radial artery occlusion rates for each group in the original FAS. **B** shows the radial artery occlusion rates for each group after imputing missing values. **C** shows the radial artery occlusion rates for each group in the PPS. **D** shows the radial artery occlusion rates for each group in the ATS. **E** shows the radial artery occlusion rates at 30-day follow-up in the original FAS. FAS = Full Analysis Set, PPS = Per Protocol Set, ATS = As-Treated Set, dTRA = distal transradial access, TRA = Transradial access, RAO = radial artery occlusion

In the FAS, RAO occurred in only 0.5% (1/213) of patients in the dTRA group, compared to 8.7% (18/212) in the TRA group with a statistically significant difference (*p* < 0.001). The achieved power was calculated based on the final sample size (dTRA group = 213, TRA group = 212), and is approximately 87.3%. Sensitivity analyses provided further confirmation of this result. In the PPS, no cases of RAO were observed in the dTRA group (0.0%), while 8.1% (15/186) of patients in the TRA group experienced RAO (*p* < 0.001). The absolute risk difference was − 7.8% (-8.0%, -3.4%), with a *P* value of < 0.001. Similarly, the ATS demonstrated an RAO rate of 0.0% in the dTRA group versus 8.0% (16/199) in the TRA group (*p* < 0.001). It was also showed an absolute risk difference of -7.8% (-8.0%, -3.4%), with a *P* value of < 0.001. Even under worst-case imputation, which assumed that all patients lost to follow-up had developed RAO, the dTRA group still exhibited a significantly lower RAO rate (3.3% vs. 11.3%, *p* = 0.0014). These findings demonstrate the distinct advantage of dTRA in reducing the incidence of early RAO following PCI. (Fig. 3A-D).

### Other secondary endpoints

#### Radial artery occlusion at 30 days

At the 30-day follow-up, the dTRA group continued to show a sustained advantage in RAO reduction. In the FAS, RAO occurred in 1.6% (3/193) of patients in the dTRA group, compared to 8.2% (16/194) in the TRA group (*p* = 0.0036). This finding underscores the long-term benefits of dTRA in preserving radial artery patency (Fig. 3E).

#### Access crossing

The rate of access site crossover was higher in the dTRA group compared to the TRA group. Specifically, crossover occurred in 15 patients (7.0%) in the dTRA group, of which 13 patients were switched to the TRA approach, primarily due to challenges in successfully cannulating the distal radial artery or maintaining guide catheter stability during the procedure. In contrast, crossover occurred in 4 patients (1.9%) in the TRA group, of which 3 patients were switched to the dTRA approach, primarily due to difficulty in puncture. Two cases in the dTRA group and one case in the TRA group that underwent crossover ultimately had puncture sites at the brachial artery and femoral artery, respectively (neither radial nor distal radial artery). The difference in crossover rates between the two groups was statistically significant (*p* = 0.0167) (Fig. 2).

#### Hemostasis time

Hemostasis time at the access site was significantly shorter in the dTRA group (3.0 h [3.0–4.0]) compared to the TRA group (6.0 h [6.0–12.0], *p* < 0.001) (Table 4). Bleeding after hemostasis occurred in 2 patients (0.9%) in the dTRA group and 1 patient (0.5%) in the TRA group.

**Table 4:** Analysis results of postoperative observation of participants

	dTRA Group (*N* = 213)	TRA Group (*N* = 212)	*P* Value
*Hemostasis duration*			
Hemostasis time (hour, IQR)	3.00, 3.00–4.00	6.00, 6.00–12.00	<0.001
Bleeding after hemostasis (N, %)	2, 0.9%	1, 0.5%	1.000
*Fugl-Meyer motor function scores (24 H post procedure)*			
Wrist score	9.98 ± 0.34	9.94 ± 0.48	0.414
Finger score	20.00 ± 0.00	19.83 ± 1.21	0.048
Total score	29.93 ± 0.76	29.82 ± 1.47	0.358
*Fugl-Meyer motor function scores (30d post procedure)*			
Wrist score	10.08 ± 1.07	9.97 ± 0.36	0.205
Finger score	19.98 ± 0.28	19.94 ± 0.72	0.511
Total score	29.98 ± 0.28	29.81 ± 1.80	0.205
*Pain and patient comfort*			
Visual Analog Scale (VAS) (N, %)			0.286
No pain	208, 97.7%	202, 95.3%	
Mild pain	3, 1.4%	8, 3.8%	
Moderate pain	2, 0.9%	2, 0.9%	
Severe pain	0	0	
Excruciating pain	0	0	
*Hospital stay and costs*			
Hospital stay (Day, IQR)	2.00, 2.00–3.00	2.00, 2.00–3.00	0.917
Hospitalization costs (USD, IQR)	3,645.52, 2,953.16-5,222.02	3,799.93, 3,085.30–5,034.60	0.680

#### Hand function recovery

At 24 h, the dTRA group showed better hand function scores compared to the TRA group (finger score: 20.00 ± 0.00 vs. 19.83 ± 1.21, *p* = 0.048). At 30 days, Differences in hand function between groups were no longer significant (Table 4).

### Pain and patient comfort

Visual analog scale (VAS) scores (Table 4) for pain at 24 h were comparable between groups, with the majority of participants reporting no pain. The incidences of mild and moderate pain were higher in the TRA group, although the differences were not statistically significant (*p* = 0.286).

### Hospital stay and costs

The median length of hospital stay was comparable between the two groups, with both the dTRA and TRA groups having a median duration of 2.0 days (IQR 2.0–3.0, *p* = 0.917). In terms of hospitalization costs, the dTRA group demonstrated a trend toward lower total costs, with a median of 3,642.52 USD (2,953.16–5,222.02) compared to 3,799.93 USD (3,085.30–5,034.60) in the TRA group. However, this difference was not statistically significant (*p* = 0.680) (Table 3).

## Discussion

This randomized controlled trial conducted in China specifically evaluates the safety and efficacy of dTRA for PCI, rather than for coronary angiography alone. Our study reflects real-world clinical practice in China, particularly the use of longer hemostasis protocols. The results demonstrated that dTRA is superior to TRA in reducing RAO at both 24 h and 30 days after PCI. In addition, the study provided a comprehensive evaluation, including assessments of RAO, hand function recovery, procedural metrics, and hospital costs. Notably, dTRA significantly reduced hemostasis times and led to modest improvements in hand function recovery at 24 h, without increasing procedural risks or costs.

### Reduction in radial artery occlusion

In this study, RAO was selected as the primary endpoint because it remains the most clinically meaningful access-related complication, irrespective of puncture site. Although dTRA is performed distally, the sheath still traverses a portion of the proximal radial artery, where endothelial injury can occur. Therefore, it is essential to determine whether the shorter sheath-artery contact segment in dTRA truly translates into a lower risk of RAO in the PCI population. Furthermore, dTRA offers several advantages, including a shorter hemostasis duration, the ability to achieve patent hemostasis through dual blood supply, and earlier mobilization. These features may indirectly reduce thrombogenicity and improve vascular outcomes. Evaluating RAO within this study allows for a comprehensive assessment of whether these theoretical benefits result in measurable vascular protection.

The primary finding of this study is the significant reduction in RAO associated with dTRA compared to TRA. At 24 h, the RAO rate was 0.5% in the dTRA group, significantly lower than the 8.7% observed in the TRA group. This reduction in RAO persisted at 30 days, with rates of 1.6% in the dTRA group versus 8.2% in the TRA group. These results are consistent with previous studies, which have demonstrated lower RAO rates with dTRA compared to TRA [3, 5]. Most notably Tsigkas et al. examined a larger cohort of 1042 patients which underwent either diagnostic coronary angiography or intervention. In that study the reduction in occlusion rate in the distal group was of similar magnitude (3.7% vs. 7.9%, respectively; *p* = 0.014) [16]. However, most prior studies included mixed populations undergoing either coronary angiography or PCI, whereas this study specifically focused on PCI patients. This distinction makes our findings particularly important, as it confirms the benefit of dTRA in a high-risk cohort. In this study, the incidence of RAO in the TRA group was slightly higher than previously reported. We speculate that this may be because our cohort included only patients undergoing elective PCI, who are at higher risk of RAO due to prolonged sheath retention despite receiving higher doses of heparin. Moreover, within the TRA group, 35.8% of patients had triple-vessel disease and 22.2% had chronic total occlusions. These complex coronary lesions increased procedural difficulty and often required the use of larger-bore sheaths, which may have contributed to the higher incidence of RAO. In our study, a total heparin dose of 100 U/kg was administered during PCI. The half-life of heparin ranges from approximately 30 min to 2 h. Clinically, a substantial reduction in heparin activity is typically observed after 4–6 h, corresponding to roughly 3–5 half-lives [17]. However, post-procedural hemostasis for radial artery interventions rarely takes less than 4 h. Furthermore, despite extending hemostasis to 6 h, some patients still required prolonged hemostasis due to uncontrollable bleeding. These factors collectively contribute to the higher incidence of RAO.

The reduction in RAO observed with dTRA is closely related to its inherent anatomical advantages. The distal radial artery is smaller in diameter and more superficial, allowing for faster hemostasis with less compression force [18]. Moreover, dTRA enables patent hemostasis due to the dual blood supply provided by the deep palmar arch. This facilitates the use of shorter hemostasis times, which has been shown to be a key factor in reducing RAO. In our team’s previous study, removing the compression 4 h after distal transradial access was found to be safe [19]. In this study, the median hemostasis time was 3.0 h in the dTRA group, compared to 6.0 h in the TRA group. This shorter hemostasis time likely contributed to the significant reduction in RAO observed in the dTRA group. Although distal transradial access significantly reduces postoperative hemostasis time, additional methods can be combined to further shorten this period. In a recent study published by our team, we demonstrated that, in patients undergoing coronary angiography via distal transradial access, administering protamine intravenously postoperatively reduced hemostasis time to 1.6 h without any complications [20, 21]. Currently, there have been no similar attempts in PCI patients. Moving forward, we plan to explore various strategies combined with distal transradial access to further minimize hemostasis time.

### Procedural safety and success

Despite the slightly higher technical difficulty associated with dTRA, as reflected by the increased number of puncture attempts, the procedural success rate was comparable between the two groups. This demonstrates that dTRA is a viable and effective alternative to TRA for PCI. Furthermore, no significant differences were observed in radiation exposure, contrast volume, or procedural time between the two groups, indicating that dTRA does not increase operator workload or patient burden.

The safety profiles of the two approaches were excellent, with no major access-related complications in the dTRA group. While two serious adverse events occurred in the TRA group, these were related to procedural complexity rather than the access site itself. Minor hematomas were rare and comparable between groups, and no cases of pseudoaneurysm, arteriovenous fistula, or major bleeding were observed. These findings underscore the safety of dTRA, even in patients undergoing complex PCI procedures.

### Hand function recovery and patient comfort

This study is the first trial to evaluate hand function in patients following dTRA and TRA PCI. Hand function recovery, as assessed by the Fugl-Meyer motor function scale [15], was better in the dTRA group at 24 h, with significant differences in finger function scores. Therefore, the early improvement in hand function recovery with dTRA may have important implications for patient satisfaction and comfort. Additionally, the shorter hemostasis time associated with dTRA not only reduces the risk of RAO but also improves patient comfort. This may enhance the overall patient experience and encourage the adoption of dTRA in clinical practice.

### Technical challenges and operator considerations

While the benefits of dTRA are clear, the approach does present some technical challenges. In our study, the rate of access crossover was significantly higher in the dTRA group (6.1% vs. 1.4%, *p* = 0.021), primarily due to difficulties in cannulating the distal radial artery or maintaining guide catheter stability. This rate is comparable to findings from prior large dTRA trials, including the DISCO RADIAL trial (7.4%) [22] and the KODRA trial (6.7%) [9]. Notably, most crossover events involved conversion from unilateral dTRA to ipsilateral TRA, allowing preservation of upper-extremity vascular access and facilitating continuation of the planned PCI without the need for alternative access sites. However, based on our findings and extensive procedural experience, distal radial artery intervention remains feasible for the majority of patients. A study in a Chinese population reported that DRA access is suitable when the distal radial artery diameter is ≥ 1.7 mm [23]. Accordingly, we adopted 1.7 mm as the minimum diameter threshold in this study, consistent with current clinical practice.

Considering the puncture difficulties of the distal radial artery, adequate technical training is critical for operators. Previous studies indicate that operators typically attain procedural proficiency after completing over 50 puncture attempts. Training programs focused on dTRA-specific techniques, such as ultrasound-guided puncture and sheath insertion, may help reduce crossover rates and improve procedural success [9, 12]. 

### Study limitations

As a single-center trial, the generalizability of our findings may be limited. Multicenter studies in China with larger sample sizes are needed to confirm our results. Additionally, the distal radial artery presents greater puncture difficulty. To align with real-world practice in China, our study employed a blind puncture approach. However, real-time ultrasound guidance can be used in challenging cases and may further facilitate the broader adoption of dTRA interventions. Future studies should explore the impact of routine ultrasound-guided puncture on procedural success and safety outcomes.

### Clinical implications and future directions

The findings of this study have important clinical implications. By significantly reducing RAO while maintaining procedural safety and efficacy, dTRA offers a superior alternative to TRA for PCI. The shorter hemostasis time and better early hand function recovery associated with dTRA further enhance its advantages, making it a more patient-friendly approach. As operator experience with dTRA grows and training programs become more widespread, the technical challenges associated with the approach are likely to diminish.

Future research should focus on multicenter, randomized trials to validate our findings in broader patient populations and explore strategies to optimize dTRA techniques. Additionally, cost-effectiveness analyses comparing dTRA and TRA are warranted, as the shorter hemostasis times and lower RAO rates associated with dTRA may translate into reduced healthcare costs over time.

In conclusion, in patients undergoing PCI, dTRA significantly reduces the incidence of RAO at both 24 h and 30 days and shortens hemostasis time compared to TRA, while improving early hand function. As a reflection of real-world clinical practice in China, our study provides robust evidence supporting the use of dTRA as a safe, effective, and patient-friendly alternative to TRA, representing a significant advancement in vascular access for coronary interventions.

## Supplementary Information

Supplementary Material 1.

Supplementary Material 2.

## Data Availability

The datasets analyzed during the current study are available from the corresponding author upon reasonable request. The study protocol and statistical analysis plan are provided in corresponding reference [13].

## Abbreviations

dTRA: Distal Transradial Access
TRA: Transradial Access
RAO: Radial Artery Occlusion
PCI: Percutaneous Coronary Intervention
FAS: Full Analysis Set
PPS: Per Protocol Set
ATS: As-Treated Set
CTO: Chronic Total Occlusion
ISR: In-Stent Restenosis
eGFR: Estimated Glomerular Filtration Rate
ACT: Activated Clotting Time
VAS: Visual Analog Scale
LVEDD: Left Ventricular End-Diastolic Diameter
LVEF: Left Ventricular Ejection Fraction
LA: Left Atrium
BMI: Body Mass Index
CABG: Coronary Artery Bypass Grafting
