# Supplementary material for: Distal transradial access for percutaneous coronary intervention: a single-center randomized controlled study (DRAGON study)
Source: BMC Cardiovasc Disord. 2025 Dec 23;26:77. doi: 10.1186/s12872-025-05456-3 (PMC12836850; doi:10.1186/s12872-025-05456-3)
Supplement: Supplementary file 1 — Supplementary Material 1. [file 12872_2025_5456_MOESM1_ESM.docx]

**Suppl Table 1. Fugl-Meyer motor function scale**

| **Body Part** | **Motor Function Test** | **Scoring Criteria** | **Preoperative** | **24H Postoperative** | **30-Day Postoperative** |
| --- | --- | --- | --- | --- | --- |
| **Wrist** | | | | | |
| Wrist Stability | (1) Elbow at 90°, shoulder at 0° | 0 points: Unable to dorsiflex the wrist to 15°.  1 point: Able to dorsiflex the wrist but unable to resist.  2 points: Able to maintain dorsiflexion against slight resistance. |  |  |  |
|  | (2) Wrist flexion and extension with elbow at 90° and shoulder at 0° | 0 points: Unable to move voluntarily.  1 point: Unable to move the wrist through the full range of motion.  2 points: Able to move smoothly and continuously. |  |  |  |
|  | (3) Scoring same as (1), with elbow at 0°and shoulder at 30° | Same as (1) |  |  |  |
|  | (4) Scoring same as (2), with elbow at 0° and shoulder at 30° | Same as (2) |  |  |  |
|  | (5) Circular motion of the wrist | 0 points: Unable to perform.  1 point: Motion is incomplete or laborious.  2 points: Normal circular motion. |  |  |  |
|  | **Subtotal (Wrist)** | |  |  |  |
| **Hand** | | | | | |
| Hand Movements | (1) Collective flexion of fingers | 0 points: Unable to flex fingers.  1 point: Able to flex fingers but not completely.  2 points: Fully able to flex fingers (compared with non-affected side). |  |  |  |
|  | (2) Collective extension of fingers | 0 points: Unable to extend fingers.  1 point: Able to relax actively flexed fingers.  2 points: Fully able to extend fingers actively. |  |  |  |
|  | (3) Grip strength 1:  Metacarpophalangeal joint extended with proximal and distal joints flexed; grip strength tested against resistance. | 0 points: Unable to maintain required position.  1 point: Weak grip strength.  2 points: Strong grip able to resist considerable force. |  |  |  |
|  | (4) Grip strength 2: Thumb adduction with all joints in the neutral position. | 0 points: Unable to perform.  1 point: Able to pinch a sheet of paper with the thumb but unable to resist pulling.  2 points: Able to firmly hold the sheet of paper. |  |  |  |
|  | (5) Grip strength 3: Thumb and index finger pinch a pencil. | Same scoring as Grip Strength 2. |  |  |  |
|  | (6) Grip strength 4: Able to hold a cylindrical object. | Same scoring as Grip Strength 2 and 3. |  |  |  |
|  | (7) Grip strength 5: Able to hold a spherical object. | Same scoring as Grip Strength 2, 3, and 4. |  |  |  |
| Hand coordination and speed: Finger-to-nose test (5 rapid consecutive repetitions). | (1) Tremor | 0 points: Marked tremor.  1 point: Mild tremor.  2 points: No tremor. |  |  |  |
|  | (2) Dysmetria | 0 points: Marked or irregular dysmetria.  1 point: Mild and regular dysmetria.  2 points: No dysmetria. |  |  |  |
|  | (3) Speed | 0 points: Slower than unaffected side by ≥6 seconds.  1 point: Slower than unaffected side by 2–5 seconds.  2 points: Difference between sides <2 seconds. |  |  |  |
|  | **Subtotal (Hand)** | |  |  |  |
|  | **Total Score** | |  |  |  |

**Reference:** A. R. Fugl-Meyer, et al. *Scandinavian Journal of Rehabilitation Medicine.* 1975;7(1):13–31.
